# Supplementary material for: Effect of Caloric Restriction on BMI, Gut Microbiota, and Blood Amino Acid Levels in Non-Obese Adults
Source: Nutrients. 2020 Feb 27;12(3):631. doi: 10.3390/nu12030631 (PMC7146580; doi:10.3390/nu12030631)
Supplement: Supplementary file 1 [file nutrients-12-00631-s001.zip › Final——Supplementary Figure-nutrients-716058.docx]

**Figure S1**

S1: Baseline gut microbial differences between two enterotype groups.


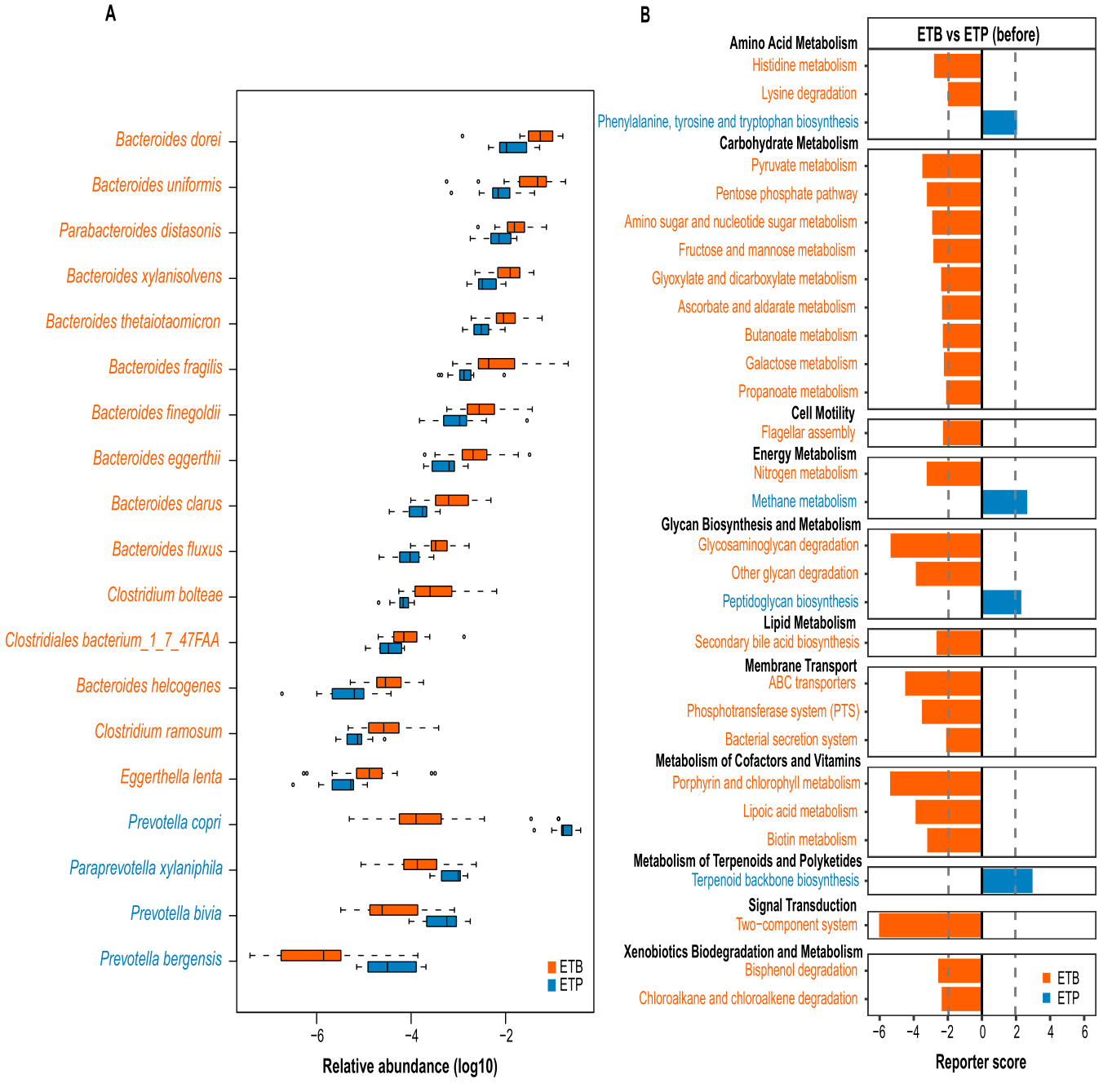


**Figure S1.** (A) Differentially enriched species between two enterotype groups. Orange and blue represent species overrepresented in ETB and ETP subjects, respectively. (B) Differential enrichment of KEGG pathways between ETB and ETP subjects. Dashed lines indicate the absolute Z score of 1.96, corresponding to 95% confidence in a normal distribution. Orange and blue bars indicate reporter scores of selected KEGG pathways overrepresented in ETB and ETP subjects, respectively.

**Figure S2**

S2: Comparison of blood amino acid levels between ETB and ETP subjects after the intervention.

**
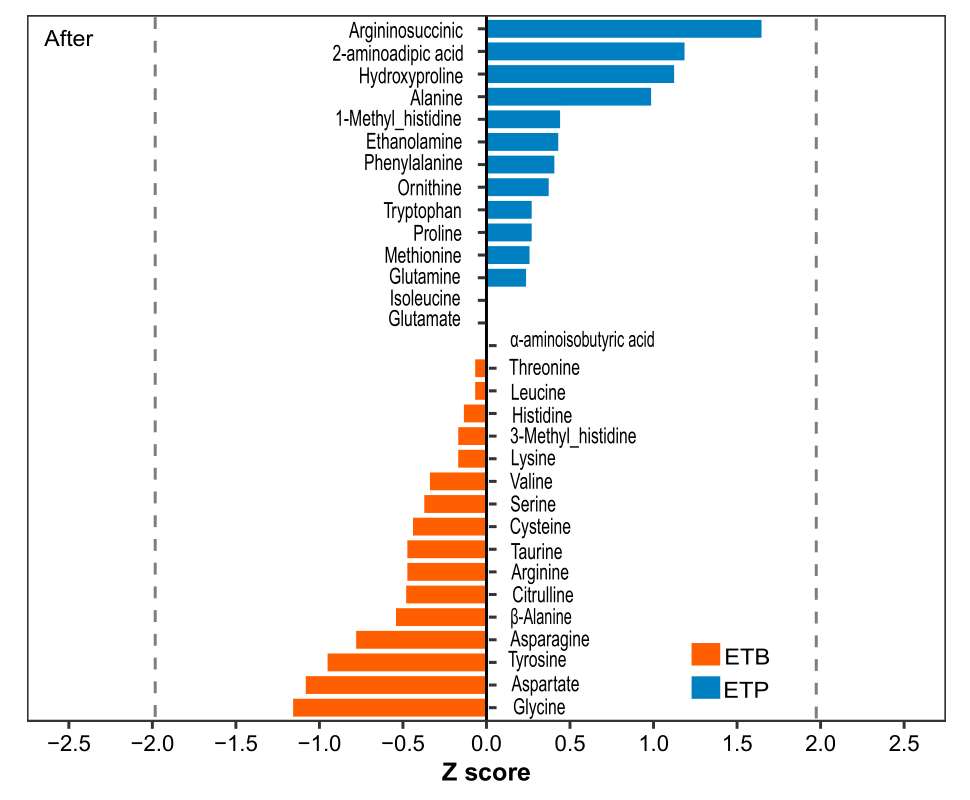
**

**Figure S2.** Comparison of blood amino acid levels between ETB and ETP subjects. Dashed lines indicate the absolute Z score of 1.96 (p = 0.05). Orange and blue bars indicate Z-score of blood amino acid overrepresented in ETB and ETP subjects, respectively.

**Figure S3**

S3: Performance of baseline BMI for prediction of BMI loss ratio.


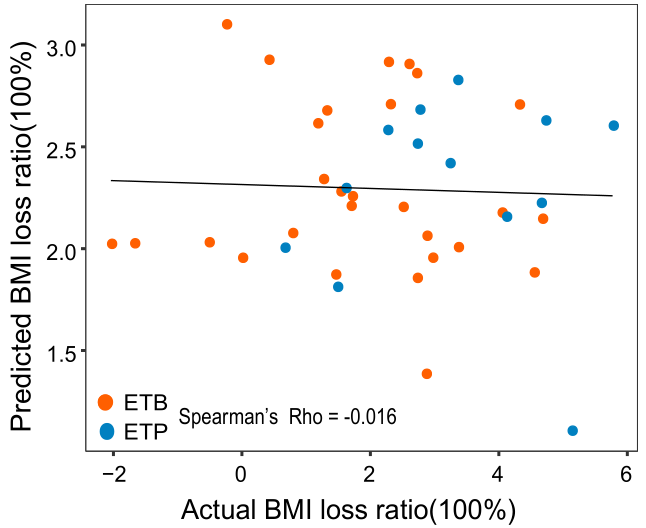


**Figure S3.** Scatter plot showing prediction performance of BMI loss ratio using baseline BMI values. A Spearman’s rho between actual BMI loss ratios and predicted BMI loss ratios was -0.016. Orange circles, ETB individuals; blue circles, ETP individuals.
